# Case report: Septic shock associated with *Granulicatella adiacens* pneumonia in a polytrauma patient following road traffic accident: rare pathogen in critical care

**DOI:** 10.3389/fmed.2026.1879098

**Published:** 2026-10-01

**Authors:** G. K. Megha, Sivadhareeni K., Mahadevaiah Neelambike Sumana, Preethi R., Deepashree Rajshekar, Supreeta R. Shettar, Yogeesh D. Maheshwarappa, Veerabhadraswamy G. S., Chinchana S. Eshwarappa, Shruthi Shree S. C.

**Affiliations:** Department of Microbiology, JSS Medical College & Hospital, JSS Academy of Higher Education and Research, Mysore, India

**Keywords:** *Granulicatella adiacens*, nutritionally variant streptococci, polytrauma, road traffic accident, septic shock, ventilator-associated pneumonia

## Abstract

**Background:**

Ventilator-associated pneumonia (VAP) is a common and serious consequence of prolonged mechanical ventilation in critically ill polytrauma patients, especially those with underlying comorbidities such as diabetes mellitus. Although VAP is often caused by gram-negative bacilli and Staphylococcus aureus, infection due to fastidious organisms is uncommon and may delay microbiological diagnosis. *Granulicatella adiacens*, formerly categorized among the nutritionally variant streptococci (NVS), is a fastidious commensal of the oral and genitourinary tract that is most commonly associated with infective endocarditis and bacteremia, whereas pulmonary infections are exceptionally rare.

**Case presentation:**

We present a case report of a 50-year-old diabetic male who sustained severe head, thoracic, and skeletal injuries in a high-impact two-wheeler RTA. He acquired ventilator-associated pneumonia with progression to septic shock on the ICU course. The initial cultures (blood, urine, ET aspirate) were sterile, but the following bronchoalveolar lavage *G. adiacens* as the predominant organism on enriched media, identifying it as the probable etiological agent. A repeat blood culture, obtained on day 4 because of suspected sepsis, subsequently also grew *G. adiacens* after the patient's death. The patient, in spite of mechanical ventilation, vasopressors, renal replacement therapy, and broad-spectrum antibiotics, developed multi-organ dysfunction and succumbed on ICU day 5.

**Conclusion:**

The case brings to the fore the potential possibility of *G. adiacens* to cause fatal opportunistic infections in polytrauma victims when invasive ICU therapy and metabolic derangements converge. Early suspicion, prompt interventions like BAL sampling, and enriched media or molecular diagnostics can facilitate the detection of fastidious pathogens in culture-negative sepsis, thus facilitating therapeutic targeting and optimizing outcomes.

## Highlights

- Polytrauma from road traffic accidents (RTAs) predisposes patients to nosocomial infections due to disrupted anatomical barriers, invasive ICU procedures, and trauma- induced immunosuppression.
- *Granulicatella adiacens*, a nutritionally variant streptococcus (NVS), is usually a commensal organism but can cause severe invasive infections—most classically endocarditis—and rarely pneumonia in critically ill or immunocompromised hosts.
- The organism's fastidious growth requirements (pyridoxal dependence) often result in initial false-negative cultures or delayed identification, complicating early diagnosis and targeted therapy.
- Diabetes mellitus and metabolic derangements (such as ketoacidosis and acute kidney injury) further impair host immunity, increasing susceptibility and worsening prognosis in such infections.
- Early bronchoalveolar lavage (BAL) sampling and use of enriched media or molecular diagnostics (16S rRNA PCR, MALDI-TOF) are critical for timely detection of fastidious organisms like *G. adiacens*.
- Empirical broad-spectrum antibiotics may not cover NVS species, so early suspicion and prompt escalation to appropriate agents are vital when deterioration occurs despite standard therapy.
- This case highlights the need to consider rare, fastidious pathogens in culture-negative sepsis among trauma patients to improve outcomes and reduce ICU mortality.

## Introduction

Globally, Road traffic accidents (RTAs) are one of the primary contributors to trauma-related morbidity and mortality. As per the WHO “*Global status report on road safety-*−*2018”*, RTAs notably share a high proportion of emergency hospital admissions and ICU utilization, especially in low- and middle-income economies (1). Patients with high-impact RTAs often present with polytrauma—a combination of head, thoracic, abdominal, and skeletal injuries— that usually requires mechanical ventilation, invasive monitoring, chest drainage, central venous catheterization, and extended ICU stay. Although these interventions are life-saving, they also promote a very permissive environment for nosocomial and opportunistic infections (2).

One such unnoticed but opportunistic pathogen is *Granulicatella adiacens*. Formerly classified among the nutritionally variant streptococci (NVS), *G. adiacens* is a Gram-positive coccus that is fastidious in nature and a normal inhabitant of the oral, gastrointestinal, and genitourinary tracts (3). Though it exists as a commensal, *G. adiacens* has been progressively involved in severe invasive infections, particularly infective endocarditis, bacteremia, central nervous system infection, and prosthetic device infections, particularly among immunocompromised or critically ill subjects (4, 5).

Notably, *G. adiacens* pulmonary infections are extremely rare, with only sporadic case reports reported to the present day (6). The stringent growth needs of *G. adiacens*, such as reliance on pyridoxal (vitamin B6), frequently lead to false-negative cultures on routine media, causing delays in diagnosis and specific therapy. In the trauma, ICU and post-injury environment, where patients are immunocompromised and exposed to various invasive interventions, such delays in diagnosis can have disastrous results, such as septic shock and multi-organ failure.

Here, we report the unusual and rare occurrence of *G. adiacens*-related ventilator-associated pneumonia and septic shock in a diabetic man after severe polytrauma caused by an RTA. The case emphasizes how injury caused by RTA, combined with intensive care treatment, can predispose to atypical, fastidious pathogens such as *G. adiacens* and underlines the importance of increased clinical suspicion and newer diagnostic techniques in culture-negative sepsis in trauma patients.

### Microbiological characteristics of *Granulicatella adiacens*

*Granulicatella adiacens* is classified within the group that was previously identified as nutritionally variant streptococci (NVS), along with *Abiotrophia* species. These are fastidious Gram-positive cocci that grow in a satellite manner around other bacteria (particularly Staphylococcus aureus) on blood agar, due to their need for pyridoxal (vitamin B6) or L- cysteine for growth (3). Due to this distinct nutritional requirement, *G. adiacens* rarely grows on routine culture media and even when it grows will tend to form small, sluggish colonies, easily missed or confused with viridans group streptococci (4).

Contemporary identification necessitates enriched culture conditions or molecular techniques like enriched media, MALDI-TOF mass spectrometry, or 16S rRNA gene sequencing (5). These constraints in clinical practice can lead to false-negative routine cultures and delayed diagnosis and targeted treatment. This is a specific challenge in critically ill or trauma patients, where prompt microbiological confirmation is critical to guide antimicrobial therapy and enhance outcomes.

## Case presentation

### Trauma and initial assessment

A 50-year-old male, of moderate build, with a prior diagnosis of type 2 diabetes mellitus, was brought to the emergency department after a high-impact two-wheeler Road Traffic Accident (RTA). He was unconscious when brought to the scene and was hemodynamically unstable upon arrival.

Initial assessment found a Glasgow Coma Scale (GCS) of E1V1M1 and a non-reactive right- sided pupil, which is consistent with severe traumatic brain injury. Vital signs revealed tachycardia (130 bpm), hypertensive crisis (210/100 mmHg), and hypoxemia (SpO_2_ 88% on room air). There were multiple external injuries, of an open, displaced fracture of the foot, a fracture of the clavicle, and chest wall tenderness.

### Imaging

Radiological assessment confirmed massive polytrauma:

1. ° CT head revealed bilateral frontal and temporal contusions and diffuse sulcal subarachnoid hemorrhage.
2. ° CT thorax revealed bilateral pneumo-hemothorax, multiple rib fractures, and patchy lung consolidations.
3. ° Skeletal survey X-ray had displaced fractures of the clavicle and foot.

These were findings that confirmed severe combined head, thoracic, and skeletal injuries with high risk for secondary complications.

### ICU course

Following a physical assessment, the patient was quickly transferred to the ICU ward, where he was immediately intubated and placed on mechanical ventilation. Bilateral intercostal chest drain tubes were inserted for pneumo-hemothorax. He needed sedation, osmotherapy (mannitol for elevated intracranial pressure), vasopressor support (noradrenaline and vasopressin), and insulin infusion for stress-induced hyperglycemia and hemodynamic stability.

Despite initial stabilization, he deteriorated with progressive systemic derangements:

- Acute kidney injury (creatinine rose from 1.95 to 3.2 mg/dl)
- Hyperkalemia (K^+^ 6.1 mmol/L)
- Positive urine ketones (suggestive of diabetic ketoacidosis)
- Falling hemoglobin (11.6 → 8.4 g/dL; internal bleeding suspected)
- Thrombocytopenia (platelets declined from 2.72 → 1.54 lakh/μl)

Continuous slow low-efficiency dialysis (SLED) was then initiated for deteriorating renal function and metabolic derangements.

### CPIS scoring for pneumonia suspicion

The patient had polytrauma and respiratory compromise at arrival after accident, necessitating mechanical ventilation and intubation. With no leukocytosis, mild non-purulent tracheal secretions, bilateral pneumothorax, severe lung contusions on CXR, and no cultures sent, the patient was initially afebrile (98–99^°°^F). The CPIS score of 2 indicated a low risk of pneumonia. By Day 2, the patient had a low-grade temperature (100–101^°°^F), increasing tracheal secretions, rising WBC, persistent hypoxia (PaO_2_/FiO_2_ ≤ 240), and developing infiltrates on imaging. This resulted in a borderline CPIS of 6 and required strict observation. Strong suspicion of ventilator-associated pneumonia was raised by continued deterioration Day 3, which was characterized by a persistent fever (~101^°°^F), worsening leukocytosis, thick purulent aspirates, further oxygenation decline, and new left lower lobe consolidation with persistent collapse. This led to the start of blood and respiratory cultures. Table 1 summarizes the clinical development during the course of the three days.

**Table 1:** CPIS development course of patient.

CPIS Parameter	Day1 admission	Score	Day 2–Early ICU	Score	Day 3–Pre–BAL	Score
Temperature	98–99°F (Afebrile)	0	100–101°F (Low–grade fever)	1	~101°F (Persistent fever)	1
WBC Count	Normal	0	Rising leukocytosis	1	Persistent leukocytosis	1
Tracheal secretions	Minimal/scanty	0	Increased, non–purulent	1	Purulent, thick secretions	2
**PaO**_**2**_**/FiO**_**2**_ **Ratio**	≤ 240 (Ventilated, hypoxic)	2	≤ 240	2	≤ 240	2
**Chest Imaging**	No infiltrates	0	Diffuse infiltrates	1	Localized consolidation (LLL)	2
Culture growth	Not sent	0	Not sent	0	Not yet collected	0
**TOTAL CPIS**		**2**		**6**		**8**
Interpretation	Low probability pneumonia		Borderline/evolving infection		High probability VAP	

Based on CPIS criteria a total CPIS of 8, which strongly indicated a high probability of ventilator-associated pneumonia.

## Antimicrobial course

The patient needed mechanical breathing after being hospitalized in serious condition. Despite being afebrile at admission, empirical intravenous cefoperazone-sulbactam (1.5 g twice daily) was started due to the possibility of ventilator-associated pneumonia (VAP) and hospital-acquired infection. By Day 2, the patient had a low-grade fever, increased respiratory secretions, and a rising Clinical lung Infection Score (CPIS), all of which were indicative of a developing lung infection. On Day 3, bronchoalveolar lavage (BAL) for microbiological culture and sensitivity testing was necessary because to continued fever, purulent endotracheal secretions, deteriorating oxygenation, and a CPIS of around 8, all of which strongly supported VAP. While awaiting the findings of the culture, empirical cefoperazone-sulbactam treatment was maintained. On day 4 blood samples collected for blood culture. Due to rapid clinical deterioration, formal antibiotic de-escalation could not be achieved (Table 2).

**Table 2:** Antimicrobial course of patient.

**Day**	Clinical status	Antibiotic given	Dose	Frequency	Purpose	Duration
Day 1	Admission, afebrile, ventilated	Cefoperazone–sulbactam	1.5 g IV	Twice daily	Empirical treatment	Started
Day 2	Low–grade fever, rising CPIS	Cefoperazone–sulbactam	1.5 g IV	Twice daily	Continued empiric	Continued
Day 3 (Pre–BAL)	Fever, purulent secretions, CPIS ~8	Cefoperazone–sulbactam (continued); BAL collected	—	—	Diagnostic	Continued
Day 3 (Post–BAL & Blood culture)	Suspected sepsis	Cefoperazone–sulbactam	1.5 g IV	Twice daily	Empirical treatment	Continued
Day 4–5	Septic shock	Cefoperazone–sulbactam	1.5 g IV	Twice daily	Empirical treatment	Continued
De–escalation	Not possible	—	—	—	Clinical instability	Not done

### Microbiological workup

Upon admission, main microbiological assessments, including blood culture sent as a part of initial workup. Initial culture was sterile. Nonetheless, the patient developed fever, exacerbated leukocytosis, and significantly raised procalcitonin levels, heightening the suspicion of ventilator-associated pneumonia (VAP). Consequently, on third day of ICU admission, a bronchoalveolar lavage (BAL) specimen was obtained for comprehensive microbiological examination.

### Sample processing and cultivation

A bronchoscopic bronchoalveolar lavage was performed, sampling the left lower lobe corresponding to the new radiological consolidation on Day 3. The direct Gram smear examination identified gram-positive cocci (GPC) in pairs and short chains among inflammatory cells, few squamous epithelial cells, confirming adequate lower-respiratory-tract sampling rather than oropharyngeal contamination. The material was inoculated onto MacConkey agar, 5% Sheep Blood Agar, and Chocolate agar, according to standard respiratory specimen procedures. Plates were incubated at 37 °C under aerobic conditions, with Blood and Chocolate agar sustained in a 5–10% CO_2_ environment.

After inoculation of the sample on blood agar, that is the material is first streaked in the primary, secondary, and tertiary quadrants to acquire isolated colonies using conventional semi-quantitative streaking procedure. Following this standard streaking of the samples, a perpendicular streak of *Staphylococcus aureus* is produced across the main and secondary streak sections of the same blood agar plate.

This *Staphylococcal* streak acts as a source of NAD (V factor) and pyridoxal, which are critical growth factors for fastidious organisms such as *Haemophilus* species and nutritionally variable streptococci (NVS) including *Granulicatella* and *Abiotrophia*. These organisms typically fail to grow independently on traditional blood agar and may stay unnoticed within the thick background of regular respiratory commensals.

### Findings on culture

Following 24 h of incubation, the MacConkey agar exhibited no growth. Tiny gray, non-hemolytic colonies were found on Blood and Chocolate agar at around 10^3^ CFU/ml, developing next to the Staphylococcus aureus streak. The colonies displayed a standard satellite phenomenon, indicative of pyridoxine (vitamin B6)-dependent growth, typical of nutritionally varied streptococci. The Gram staining of the colonies reaffirmed the presence of gram-positive cocci arranged in pairs and short chains. Colony count of approximately 10^3^ CFU/ml falls below the conventional bronchoscopic BAL threshold of ≥104 CFU/ml for VAP diagnosis; however, prior empirical cefoperazone-sulbactam exposure is likely to have suppressed absolute colony counts, and the result is interpreted in conjunction with the inflammatory Gram stain, the clinical trajectory, and a CPIS of 8, all of which support clinical significance rather than colonization.

### Identification and antimicrobial susceptibility testing

Based on preliminary findings suggestive of *Streptococcus species*, following tests were performed: Optochin (OP), bacitracin (BA) sensitivity tests, and Bile-Esculin test. The isolate exhibited resistance to OP and tested negative for bile-esculin.

For conclusive identification, the isolate was processed using the VITEK-2 Compact system (GPC identification card). Vitek-2 identified the organism as *Granulicatella adiacens* with high confidence. We acknowledge that VITEK-2 has a restricted database for nutritionally variant streptococci, and that MALDI-TOF mass spectrometry or 16S rRNA gene sequencing—the preferred confirmatory methods for NVS—were not available at our center; this represents a limitation of the identification.

The Clinical and Laboratory Standards Institute (CLSI) M45 guidelines for fastidious and non-frequent bacteria say that disk diffusion methods shouldn't be used for *G. adiacens* because they don't always work; instead, broth microdilution MIC testing in cation-adjusted Mueller–Hinton broth with lysed horse blood and pyridoxine hydrochloride is suggested to get accurate results. Under these conditions, *G. adiacens* is not always sensitive to β-lactam antibiotics. It is less sensitive to penicillin and third-generation cephalosporins like ceftriaxone, but most isolates are still sensitive to vancomycin, meropenem, levofloxacin, and clindamycin.

CLSI M45 suggests broth microdilution-based MIC testing with pyridoxine-supplemented media for *Granulicatella adiacens*. However, formal antimicrobial susceptibility testing was not feasible in this instance due to constrained laboratory resources and the unavailability of specialized media and MIC panels necessary for nutritionally variant streptococci. Our laboratory does not regularly maintain lysed horse blood–pyridoxine–supplemented broth or specific NVS MIC cards, and referral testing would have caused an unacceptably long delay in starting targeted therapy. This practical approach is based on the fact that many tertiary care centers in low- and middle-income countries have to deal with real-world problems. When advanced AST facilities aren't available, management often has to use syndromic assessment and evidence-based empirical therapy.

### Blood culture

At the time of admission, the initial blood culture showed no bacterial growth. On day 4 of ICU admission, following the onset of fever, worsening leukocytosis, elevated inflammatory markers, and clinical suspicion of sepsis, a repeat blood culture was obtained. Although the patient died on day 5 before the blood culture results became available, the repeat culture subsequently yielded *Granulicatella adiacens*, which was identified by the VITEK-2 Compact system. The microbiological concordance between the respiratory and bloodstream isolates supports *G. adiacens* as the probable etiological pathogen responsible for the patient's septic illness

### Role of the pathogen (*Granulicatella adiacens*) in clinical deterioration

The fastidious nature of *G. adiacens* caused a delay in its identification, as routine culture media were initially unable to sustain its growth. Consequently, empirical broad-spectrum antibiotics (cefoperazone-sulbactam) were initiated; however, focused treatment could not be instituted promptly.

In the context of severe polytrauma, metabolic disturbances, and critical-illness–related immune impairment, *G. adiacens*—identified as the predominant respiratory isolate with microbiological concordance from a subsequent blood culture—is the most plausible contributor to the clinical deterioration. Delayed identification precluded targeted therapy, and the patient progressed to multi-organ dysfunction.

This cascade culminated in refractory hypotension and death on day 5 of ICU, in the face of maximal organ support, vasopressors, renal replacement therapy, and full-spectrum critical care interventions. The Pathophysiological mechanism of *Granulicatella adiacens* infection in polytrauma is given in Figure 1.

**Figure 1:**
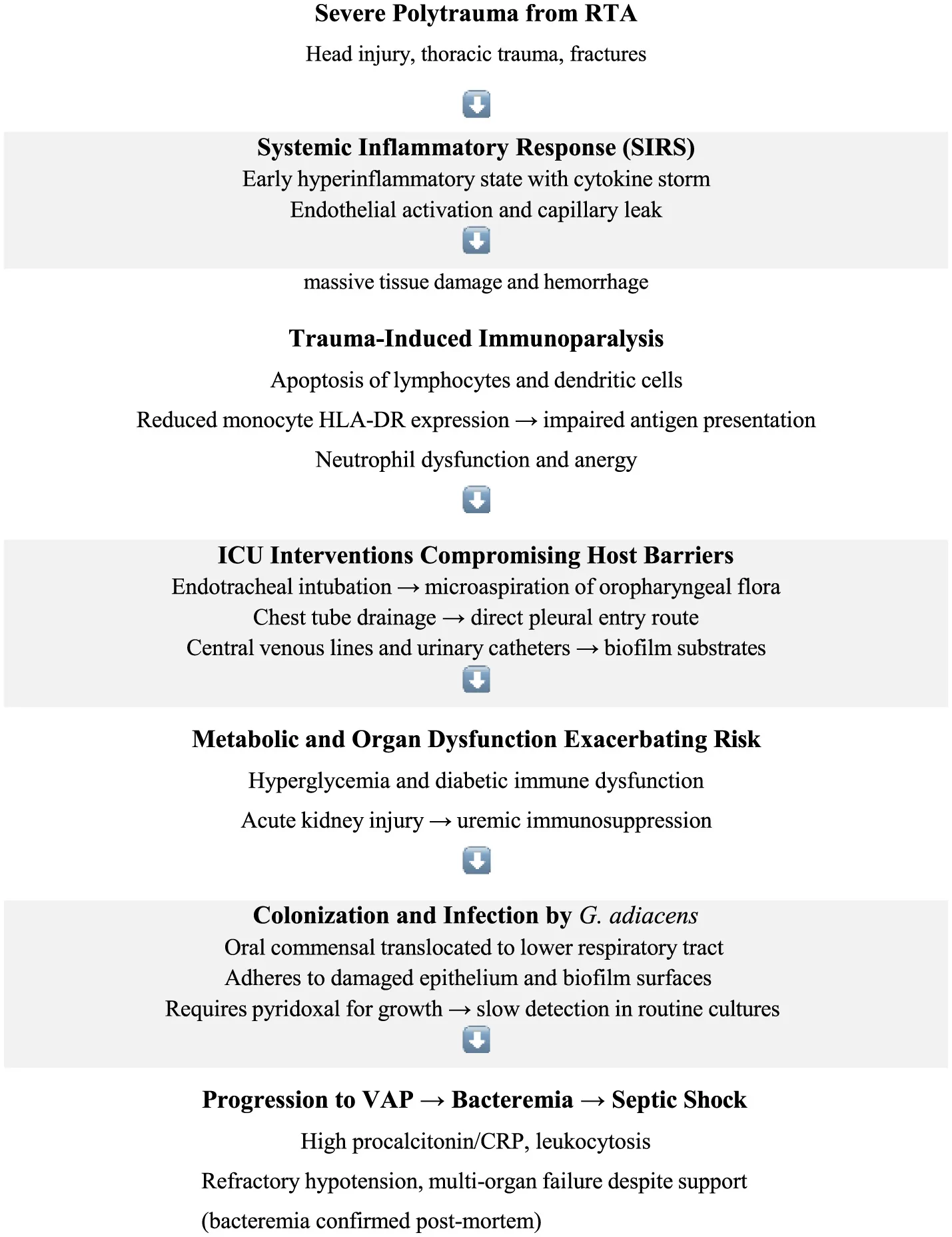
Pathophysiological mechanism of *Granulicatella adiacens* infection in polytrauma.

## Discussion

*Granulicatella adiacens*, once grouped with nutritionally variant streptococci (NVS), is an uncommon yet potentially aggressive pathogen. It is more often linked to serious conditions, including bacteremia, endocarditis, and deep-seated abscesses (7, 8). Though it is usually a commensal mucosal resident of the oral, gastrointestinal, and genitourinary tract, it can lead to opportunistic infections, especially in immunocompromised patients or after invasive medical interventions. Its infections are uncommon but can be underdiagnosed because of its fastidious growth requirements, with the study evidence that estimated, 5–6% of cases of culture-negative endocarditis are due to *Granulicatella spp* (9). Pulmonary infection is very uncommon and rare, and less than 15 cases (< 15) have been reported in the literature. This case illustrates its pathogenicity in that trauma- associated immune dysregulation and interventions in the ICU may provide a permissive environment for such fastidious organisms.

### Polytrauma and susceptibility to nosocomial infection

Severe polytrauma needs an extended stay in the intensive care unit (ICU) with mechanical respiratory support, endotracheal intubation, chest tube drainage, and central venous catheterization, all of which breach natural skin and mucosal barriers and introduce artificial surfaces that encourage microbial colonization (10, 11). Polytrauma causes severe immune dysregulation in addition to mechanical disturbance. Patients quickly move from a hyperinflammatory state (SIRS) to a compensatory anti-inflammatory state (CARS), which is marked by lymphocyte apoptosis affecting CD4^+^ and CD8^+^ T cells, neutrophil exhaustion with impaired chemotaxis and oxidative burst, and monocyte deactivation indicated by decreased HLA-DR expression (12). Even in the absence of typical immunosuppression, colonization and infection are made possible by this immunological paralysis, which weakens the host's ability to control unusual pathogens like *G. adiacens*.

### ICU interventions and breach of natural barriers

Within 48 h of intubation, endotracheal tubes form biofilms, creating a persistent microbial reservoir that promotes lower respiratory tract seeding (13). Exposure to broad-spectrum antibiotics damages local flora and creates ecological niches, where fastidious, slow-growing organisms such as *G. adiacens* might become opportunistic infections (8, 9). The colonization of *G. adiacens* in this patient was probably made easier by antibiotic-induced dysbiosis. The organism then functioned as a secondary pulmonary invader, taking advantage of impaired mucociliary clearance and epithelial damage from both trauma and extended mechanical ventilation (14, 15).

### Diabetes mellitus as a compounding factor

Aside from trauma and ICU interventions, the patient's underlying diabetes mellitus was a significant compounding factor for susceptibility to infection. Diabetes alone compromises innate immunity, mainly by the deleterious effects of hyperglycemia. Hyperglycemia suppresses neutrophil chemotaxis, phagocytosis, and intracellular killing and thus compromises the first line of defense against invading pathogens (16). Further, chronic hyperglycemia enhances non-enzymatic glycosylation of complement and immunoglobulin proteins and diminishes their functional efficiency. It also promotes enhanced bacterial adhesion to respiratory epithelium, allowing for a more permissive culture for colonization and invasion.

In the current case, these mechanisms probably added insult to the trauma-induced immune dysfunction that was already occurring in the ICU environment. The additive effect of compromised neutrophil function, diminished humoral activity, and increased bacterial adhesion pushed the scales further in the direction of opportunistic pathogens like *Granulicatella adiacens*, enabling it to proceed from colonizer to clinically relevant pulmonary pathogen.

### *Granulicatella adiacens*—an emerging but elusive pathogen

A growing number of invasive diseases, such as bacteremia, infective endocarditis, osteomyelitis, prosthetic joint infection, and deep-seated abscesses, have been linked to *G. adiacens* (8, 17). It is thought to be responsible for 5–6% of instances of culture-negative endocarditis due to its obligatory pyridoxal auxotrophy and fastidious growth requirements, which often cause false-negative or delayed cultures in typical routine labs. Adhesion to extracellular matrix proteins (fibronectin and laminin) and biofilm formation enhance virulence, allowing for resistance to host immunity and antimicrobial treatment (7, 8).

Pulmonary infections are still very uncommon and are almost exclusively reported in severely ill immunocompromised individuals as lung abscess, empyema, or spontaneous pneumonia (15, 18–20). Even in patients without traditional risk factors, the combination of low clinical suspicion, delayed culture positive, and unusual host presentation usually leads to a significantly delayed diagnosis. For prompt clinical identification and focused therapy, it is crucial to understand its possible involvement in invasive pulmonary illness.

Despite immunological dysregulation marked by neutrophil fatigue, reduced monocyte HLA-DR expression, and CD4^+^ and CD8^+^ T-cell death, trauma and severe illness further predispose individuals (12). Comorbid conditions like diabetes mellitus increase this susceptibility by glycosylating important immune proteins and hindering phagocytosis. Through endothelium adhesion and biofilm-mediated persistence, *G. adiacens* takes advantage of these host deficits, which explains why pulmonary infection may develop even in initially immunocompetent trauma patients—a presentation that is still very rare and underrepresented in the literature.

### Pathogenic contribution to clinical deterioration

Acute polytrauma in this patient caused SIRS-driven immunoparalysis, which is characterized by cytokine surge, immune cell apoptosis, impaired antigen presentation, and neutrophil dysfunction. At the same time, anatomical barriers were breached by the insertion of a chest tube, endotracheal intubation, and mechanical ventilation. Within 48 hours, biofilm development on the endotracheal tube created a chronic microbial reservoir, and administration to broad-spectrum antibiotics disturbed native flora, allowing *G. adiacens* to proliferate. By impairing neutrophil chemotaxis, phagocytosis, and uremic immunosuppression, co-occurring diabetes mellitus and renal impairment further weakened innate immunity and eliminated the host's ability to contain even low-virulence pathogens.

*G. adiacens*'s fastidious pyridoxal dependence resulted in significant delays in organism identification, allowing progression from localized VAP to systemic dissemination consistent with the post-mortem blood culture result, which clinically manifested as severe leukocytosis, refractory hypotension, and rapidly escalating inflammatory markers (12).

### Conceptual stepwise mechanism

#### Diagnostic limitations and nuances

Identification of *Granulicatella adiacens* poses a special diagnostic pitfall in critically ill patients. This is a fastidious bacterium that grows only on pyridoxal-supplemented or enriched media and tends to yield negative or delayed-positive blood cultures (21, 22). Such infections can therefore be inappropriately labeled as “culture-negative” with consequent delays in targeted therapy. In a few cases, *G. adiacens* can only develop in co-culture with helper bacteria (e.g., *Staphylococcus aureus*), appearing as the typical “satellite phenomenon” on blood agar (7). The dainty colonies go unnoticed without enriched media.

Traditional blood culture systems also have drawbacks: detectable growth can take up to 48– 72 h to appear, and cultures tend to be discarded after 5 days, with the risk of false negatives (9). Such time delays are clinically meaningful for patients in ICU, where every hour of sepsis left untreated raises mortality (10).

These limitations underscore the need for contemporary diagnostic modalities:

- 16S rRNA gene PCR and universal bacterial PCR enable direct detection of bacterial DNA from clinical specimens, avoiding culture lag (8).
- MALDI-TOF mass spectrometry can quickly and precisely identify colonies after growth has taken place (22).
- Enriched or pyridoxal-supplemented media use increases the chances of recovering the organism when suspicion is great (21).

For ICU clinicians, maintaining an early index of suspicion is critical when patients deteriorate despite broad-spectrum antibiotics and negative cultures. In such settings, rapid molecular and mass spectrometry-based diagnostics are invaluable, enabling timely identification and guiding appropriate antimicrobial therapy before irreversible septic progression occurs (12).

#### Therapeutic challenges and complexity

The management of *Granulicatella adiacens* infection is full of challenges, due to its distinctive microbiological characteristics and clinical presentation. A key characteristic is the ability to form biofilm, which provides protection against host defense and antimicrobial action, making the infection challenging to eliminate (7, 8). Adding to this is the relative resistance of the organism to β*-lactams*—a situation in which isolates are susceptible by MIC testing but are not effectively killed. This “β*-lactam tolerance*” usually requires the administration of synergistic regimens, most often the combination of a β-lactam or vancomycin with an aminoglycoside, especially in invasive illness like endocarditis (8, 20).

Antimicrobial susceptibility patterns are unreliable and volatile. Whereas *G. adiacens* is routinely said to be susceptible to penicillin, ceftriaxone, and vancomycin, instances of decreased susceptibility and absolute resistance to β-lactams and cephalosporins have been described (8, 9). Such occurrences render empiric treatment particularly challenging in ICU environments, where broad-spectrum cephalosporins or carbapenems constitute first-line treatment but could fail to offer consistent coverage for this bacterium (10). Another factor complicating treatment is slow growth and delayed diagnosis. In the time it takes for cultures to provide results, the infection may be progressed to the extent that therapeutic action is less beneficial (22). For deep-seated or biofilm-associated infections, extended combination therapy is necessary, usually 4–6 weeks in length, since shorter durations are likely to fail (20).

In our patient, initial empiric coverage with cefoperazone-sulbactam most likely provided suboptimal activity against *G. adiacens*. Before identification of the pathogen and consideration of appropriate therapy, the patient had already developed irreversible septic shock. This highlights an important lesson: in ICUs due to trauma, where patients are immunocompromised, instrumented heavily, and exposed to broad-spectrum antibiotics, early detection of fastidious organisms such as *G. adiacens* is vital for survival (12, 13).

#### Clinical implications and literature context

*Granulicatella adiacens* is a rare but increasingly recognized invasive pathogen beyond its classical association with infective endocarditis. Particularly, pulmonary infections are uncommon: in the published literature, there are fewer cases of pneumonia, lung abscess, empyema, and VAP linked to *G. adiacens* or closely related nutritionally variant streptococci (NVS), and the number of fatal outcomes in critically ill patients is still underestimated. In this modest body of information, the current case offers a significant and underrepresented context: severe polytrauma with many co-existing risk factors. Table 2 summarizes the recorded pulmonary, intensive care unit, and systemic *G. adiacens* infections in chronological order.

Mvelase et al. (17) reported thoracic empyema caused by *Granulicatella elegans*, a closely similar NVS species, in a patient with concomitant pulmonary TB and HIV infection. This is the first well-documented evidence that NVS may cause significant pleuropulmonary infection. After pleural drainage and penicillin treatment, the patient recovered, demonstrating that NVS pathogens may cause thoracic suppurative illness in immunocompromised hosts in addition to endocarditis. In the same year, *G. adiacens* was isolated from sterile bodily fluids, primarily blood cultures and a cerebrospinal fluid shunt, in seven patients, five of whom were younger than 2 years old, according to a pediatric case series from Amrita Institute of Medical Sciences, India [Krishna et al. (23)]. The organism's therapeutic significance beyond endocarditis and across age groups and anatomical compartments was confirmed by the fact that six of the seven survived targeted antibiotic treatment. Comparative summary of documented *Granulicatella adiacens* and NVS infections is given in Table 3.

**Table 3:** Comparative summary of documented *Granulicatella adiacens* and NVS infections.

Study (Author, Year)	Setting	Risk factors/comorbidities	Site of infection	Treatment	Outcome	References
Mvelase et al. (2016)—NVS thoracic empyema (G. elegans) in TB/HIV patient	Respiratory ward	Pulmonary TB, HIV infection	Thoracic empyema (G. elegans, NVS)	Pleural drainage + penicillin	Recovered	(17)
Gardenier et al. (2011)—*G. adiacens* bacteraemia in an elderly trauma patient	Trauma surgical unit	Elderly female, trauma	Bacteraemia	Targeted antibiotics	Survived	(15)
Krishna et al. (2014)—*G. adiacens* isolated from sterile body fluids: case series from India	Tertiary center; 7 cases	Predominantly pediatric; various comorbidities	Bacteraemia, CSF shunt fluid	Standard therapy (varied per isolate)	6/7 survived	(23)
Aydin Teke et al. (2025)—*G. adiacens* infections in children: single–center retrospective study	Pediatric tertiary center (12–year series)	Heart disease, malignancy, GI/neurological comorbidities	Bacteraemia, endocarditis, CRBSI	Culture–directed therapy	Mostly survived	(14)
Plate et al. (2018)—Aortic endograft infection with M. chimera and *G. adiacens*, Switzerland	Post–aortic endograft repair	Prosthetic aortic graft, polymicrobial (M. chimera co–infection)	Aortic endograft infection	Graft explantation + 12 months antibiotics	Recovered	(24)
Hosogaya et al. (2019, ATS abstract)—Multiple lung abscesses and pharyngeal abscess due to *G. adiacens*	Not specified (conference abstract)	Likely immunocompromised	Multiple lung abscesses + pharyngeal abscess (co–infection with S. constellatus)	Not specified	Recovered	(25)
Acharya et al. (2020)—Parapneumonic effusion and bacteraemia secondary to *G. adiacens* in immunocompetent host	General ward	Immunocompetent	Complicated parapneumonic effusion + bacteraemia	Ampicillin + gentamicin (targeted)	Recovered	(26)
Yang et al. (2021)—Lung abscess with *G. adiacens* and Eikenella corrodens co–infection in lung cancer patient	Adult with bronchogenic carcinoma	Bronchogenic carcinoma, immunosuppression	Lung abscess + pleural effusion (polymicrobial: *G. adiacens* + E. corrodens)	Drainage + >5 weeks antibiotics	Gradual recovery	(18)
Purohit et al. (2022)—*G. adiacens* as unusual cause of empyema	ICU/ward	Predisposing risk factors (not fully detailed)	Empyema → sepsis → necrotizing fasciitis	Aggressive broad–spectrum therapy	Fatal	(19)
Freire et al. (2022)—Atypical lung abscess due to *G. adiacens* and S. constellatus in immunocompetent patient	General ward	Immunocompetent, no classical risk factors	Lung abscess (polymicrobial: *G. adiacens* + S. constellatus)	Prolonged antibiotic therapy	Recovered	(27)
Téllez et al. (2018)—IE due to Abiotrophia and Granulicatella species: 76 cases, 2000–2015	Tertiary hospital, Spain (multicentre retrospective)	Adults with structural heart disease, prosthetic valves	Infective endocarditis	Standard IE therapy (variable duration)	Non–trivial mortality; worse with structural heart disease	(16)
**Present case (2025)**	**Surgical ICU (polytrauma, diabetic)**	**Polytrauma, type 2 diabetes, DKA, AKI, mechanical ventilation, chest tubes**	**VAP** ** → clinical sepsis** ** → MODS**	**Empirical cefoperazone–sulbactam** **+** **SLED** **+** **maximal ICU support**	**Died (refractory septic shock, ICU Day 5)**	**—**

Gardenier et al. (15) reported the first recorded case of *G. adiacens* bacteraemia in a trauma patient, an elderly woman who was accurately diagnosed and effectively treated, demonstrating the organism's relevance in trauma and device-related settings. This observation is especially relevant to the current case, where polytrauma produced a remarkably similar immunocompromised environment. Evidence of *G. adiacens* as a pathogen in prosthetic and device-associated infection was provided by Plate et al. (24), who described a polymicrobial aortic endograft infection co-involving Mycobacterium chimera and *G. adiacens*, successfully managed only after complete graft explantation and 12 months of antimicrobial therapy. The organism's ability to form biofilms and a preference for implanted surfaces were confirmed in this instance.

Aydin Teke et al. (14) conducted a 12-year single-center retrospective analysis in the pediatric domain and found that *G. adiacens* was detected in only 0.1% of positive blood cultures across presentations such as bacteraemia, endocarditis, and catheter-related bloodstream infection. Despite being uncommon, the organism was clinically relevant in all afflicted individuals, and the majority of them lived with early targeted treatment, highlighting the fact that prompt detection is the primary factor influencing prognosis. Hosogaya et al. (25) reported the first documented case of simultaneous multiple lung abscesses and pharyngeal abscess due to *G. adiacens* in a polymicrobial context, described in an ATS conference abstract.

According to Yang et al. (18), a 52-year-old lady with bronchogenic cancer had the first lung abscess linked to *G. adiacens*. The patient needed more than 5 weeks of antibiotic therapy and pleural drainage before the organism was co-isolated with Eikenella corrodens. This indicates both the organism's pathogenic potential in immunosuppressed respiratory epithelium and the length of treatment courses required. The same year, Acharya et al. (26) reported a case that stands out for its patient profile: a previously immunocompetent host who recovered with ampicillin + gentamicin after developing a complex parapneumonic effusion and concomitant bacteraemia caused by *G. adiacens*. This instance clearly relates to the current case, where the patient's immunocompetence was only acutely reduced by trauma and metabolic disruption, and it challenged the notion that *G. adiacens* infection required preexisting immunological impairment.

Together, two further 2022 studies show both extremes of the result range. The organism can occasionally cause pulmonary infection even in the absence of classical immunocompromise, as demonstrated by Freire et al.'s (27) description of a previously healthy young patient who developed an atypical lung abscess due to *G. adiacens* and Streptococcus constellatus and recovered with prolonged antibiotic therapy. In contrast, Purohit et al. (19) described a fatal case of *G. adiacens* empyema that, in spite of vigorous treatment, developed to necrotizing fasciitis and systemic sepsis, demonstrating the disastrous effects of delayed detection of this picky pathogen. Téllez et al. (16) further contextualized the scope of *G. adiacens* invasive disease beyond the lung by confirming non-trivial mortality, periannular complications, and a particularly poor prognosis in patients with prosthetic valves or structural heart disease in their multicenter analysis of 76 cases of infective endocarditis caused by *Abiotrophia* and *Granulicatella* species over a 15-year period. This organism group is also linked to high rates of therapy failure and recurrence, according to the AHA recommendations, which need combination synergistic regimens in invasive disease (20).

In considering this body of data, the present case (2025) makes a clinically unique and neglected addition. This case describes the convergence of severe polytrauma, metabolic comorbidity (type 2 diabetes, acute kidney injury, diabetic ketoacidosis), and intensive invasive ICU interventions as a sufficient permissive environment for *G. adiacens* may evolve from oral commensal to lethal ventilator-associated respiratory pathogen, in contrast to previous reports that were dominated by hematological malignancy, prosthetic device infection, congenital heart disease, or non-specific immunocompromise. The quick development of multi-organ failure and mortality in spite of broad-spectrum antibiotics and maximum organ support highlights how limited the window for successful intervention is in this situation. Therefore, this case serves as a reminder to ICU clinicians of two important points: first, that *G. adiacens* should be included in the differential diagnosis of culture-negative deterioration in any critically ill trauma patient; and second, that early bronchoalveolar lavage using enriched media and expedited molecular diagnostics may be the only practical route to identification and targeted therapy before the chance for intervention is lost.

## Conclusion

This case highlights the unusual but potentially lethal role of *Granulicatella adiacens* as an opportunistic pathogen in critically ill polytrauma patients. Though classically associated with infective endocarditis and bacteraemia, its presentation as ventilator-associated pneumonia—confirmed here by BAL culture and supported by a subsequent positive blood culture—represents an exceptionally rare clinical scenario. Our patient's trajectory shows the manner in which the confluence of high-impact trauma, invasive ICU interventions, and metabolic comorbidities (diabetes, acute kidney injury, ketoacidosis) provided a permissive environment for this fastidious organism to evolve from colonizer to pathogen.

The delayed positive culture and primary negative culture, characteristic of *G. adiacens* because of the pyridoxal requirement, also added to delayed targeted therapy, permitting progression to fulminant sepsis and multi-organ dysfunction. This emphasizes the diagnostic dilemma presented by nutritionally variant streptococci in ICU practice, where early and accurate microbiological identification is essential to survival.

Clinicians must maintain a high index of clinical suspicion for fastidious organisms such as *Granulicatella* in culture-negative sepsis in trauma patients, particularly when deterioration is unexplained. Early bronchoalveolar lavage sampling, enriched medium, and expedited molecular diagnostic methods could enhance detection and outcomes in such uncommon but fatal infections.

## Data Availability

The original contributions presented in the study are included in the article/supplementary material, further inquiries can be directed to the corresponding author.
